# Exploring the relationship between the tourist behavior and the spatial characteristics for rural tourism

**DOI:** 10.1038/s41598-025-93517-0

**Published:** 2025-03-18

**Authors:** Ka Li, YiNa Zou, Hao Wang, Shuolei Chen

**Affiliations:** College of Landscape Architecture, Nanjing Forestry University, Nanjing, 210037 China

**Keywords:** Tourist behavior, Spatial characteristic, Rural tourism, Cultural legacy, UAV, Sustainable urban and rural development, Ecology, Environmental social sciences

## Abstract

With global urbanization, rural tourism has become a thriving trend for urban-rural sustainable development in addition to the urban landscape. However, research on rural landscape planning is still lacking. The topography of the rural areas is complex, with mountains and buildings arranged in accordance with the terrain, and pedestrian data is difficult to collect. Therefore, this study adopts mixed methods to obtain high-precision data. This study aims to investigate the relationships between tourist behavior and spatial characteristics. The results indicated that (1) Different rural spaces formed an uneven distribution of tourists’ spatial-temporal behavior characteristics, which could be attributed to three potential factors: easy space accessibility, good visual permeability, and herd mentality; (2) Visual space had a strong influence on guiding tourists compared to the passable space; (3) Historical trees, heritage buildings and cultural legacy are the positive influencing cultural factors for tourist attraction in spaces. Furthermore, these findings provided rationales to mobilize the utilization of the rural landscape resources and enhance the sustainable urban-rural development. These findings and methods improve our understanding of the temporal–spatial tourist behavior in rural tourism, which is of great significance for rural tourism planning and cultural legacy protection.

## Introduction

In the face of rapid global urbanization, the expansion of urban boundaries has resulted in a slew of social and environmental issues^1^. There has been a severe shortage of urban landscape resources. The stresses of urban lifestyles have led to a “counter-urbanization” syndrome^2^. In order to provide people with enough opportunity to engage in various activities, which can be realized in rural areas. Rural tourism is significant for sustainable development under urbanization. Consequently, we have to understand people’s activities and behaviors in the rural area. There is a trend toward seeking a more natural environment. Forests and suburbs surrounding cities are becoming tourist destinations. Rural tourism has notably become one of the most popular travel activities among city residents. Rural spaces have transcended their connection with agricultural commodity production, transforming into sites that seamlessly integrate with tourism, leisure, specialty food production and consumption, and e-commerce^3^. Among these various roles, their most significant function is to serve as a prime destination for rural tourism^4^. The destination is a main characteristic of rural tourism in both developed and developing countries^5^. Rural destinations are mainly used by local tourists, and there are very few exceptions that cater to international clients^6^. China, as one of the developing countries, is no exception. Urban research has been extensively studied. However, because rural research environments differ from urban ones, they have not been as thoroughly investigated^7^. Therefore, we should start by understanding people’s tourist behavior. Rural tourism advocates for a free approach to travel, emphasizing the autonomy of tourists^8^. Unlike traditional urban parks that are limited to specific and predetermined tour routes, rural tourism encourages travelers to chart their own path, aligning with the principles of experiential landscape design, which aim to create flexible, visitor-driven environments^9^. The spatial morphology of the countryside is also complex^10^. Exploring the relationship between tourist behavior and rural spatial characteristic is crucial for the development of rural tourism.

## Theoretical background and hypothesis development

### Understanding tourists’ behavior is fundamental to promote rural tourism

The study of tourists’ behavior plays a vital role in exploring the attraction and development of tourism. Presently, numerous approaches have been developed, some in conjunction with traditional methods. Among them, the most direct and effective approach involves recording tourists’ travel routes and times using paper map diaries or questionnaires^11^. Questionnaires are widely employed to study tourist behavior^12–16^. For instance, researchers Lin et al. used the survey data collected from Dafu Mountain Forest Park in China to evaluate tourists’ questionnaires about urban forest parks, aiming to improve tourists’ leisure experiences and satisfaction^17^. However, these traditional methods demand significant time investments from researchers to collect and organize data samples. Furthermore, due to the impracticality of recording the complete travel track of tourists and the limitation of small sample data, these methods can result in reduced research efficiency.

With the advancement of computer technology, it has become prevalent in tourism research. Presently, tourism behavior analysis based on big data has become a widespread trend, and is regarded as an effective and efficient tool for tourism planning. Presently, tourist behavior analysis is based on big data, including three primary categories: user-generated content (UGC), device data and transaction data^18^. Computer technology can be used to collect data about tourists during the journey in a more convenient and efficient manner compared to traditional data measurement methods, for such data does not demand any additional time or resources from the participants^19^.

As a result, UGC data, specifically online text, and online photos, has progressively become a favored data source for the practical study of tourist behavior. In comparing the UGC of tourists in China and Spain, researchers have drawn the conclusion that empowerment has the greatest impact on UGC participation in China, but in Spain, co-creation is the main source of incentive^20^. The feasibility of utilizing images photographed by tourists to recognize tourist behavior has been verified in a few research investigations. For instance, researchers have proposed employing computer technology to identify visitor preferences by analyzing images of scenic spots and food^21,22^.

Device data, including global positioning systems (GPS) data, mobile roaming data, Bluetooth data, and so on is widely used to study tourist behavior. GPS data and Bluetooth data are extensively utilized for this purpose. Researchers frequently utilize portable GPS data loggers to capture detailed information about the locations visited by visitors^23^. Radio-frequency identification (RFID) systems are also applied to study tourist behavior. These systems have been effectively utilized in numerous theme parks, including Dolly’s (TN), Steamboat Ski Resort (CO), Wild Rivers (CA), and Legoland Amusement Park (Denmark)^24,25^. Eye trackers are increasingly employed in a growing number of studies to analyze and draw elements that influence people’s visual behavior and satisfaction preferences in woodland leisure. Although an eye tracker can tell us what the tourist is watching, it cannot provide information on why or how they feel after viewing it^26^.

Transaction data, including web search data, webpage visiting data and online booking data, can be downloaded on the internet. In a study conducted during the period of the COVID-19 pandemic, researchers analyzed 33 destinations representative of various types of tourism through credit card transaction data. According to the study, the sense of danger and the destination’s proximity to home may account for variations in tourist behavior among different locations. Tourists tend to show a preference for scenic areas and enticing beach cities^27^. By utilizing the concept of the gravity model, transaction data has even been used to understand seasonal differences in tourist behavior, specifically in terms of the number of inquiries gained through an official website^28^.

Recently, due to the impact of the COVID-19 pandemic and the high cost of traditional GPS data recorders, there has been a shift in the method of collecting GPS data. Some scholars have utilized tracking apps installed on mobile devices to collect data. The trajectory data obtained from open global positioning systems has proved to be more efficient compared to the standard self-collected GPS data^29^.

In conclusion, extensive research has been conducted on big data analysis of tourist behavior, and the study has focused on different tourist’s dwell spaces. In the city, researchers have explored various areas of tourist’s dwell spaces, such as museums^30^, theme parks^31^, zoos^32^, etc. However, the study of tourists’ behavior is widely used in urban and regional tourism research^33–35^, while it is rarely used in rural tourism research, with limited research addressing actual tourist behavior data. The reasons are as follows: (1) The data on tourist behavior in rural areas is not accurate. (2) The scale of rural areas is relatively large. (3) The population distribution is more random. The methods and tourist behavior are very different from those in urban areas. Therefore, we need to use the methods of urban tourist behavior analysis to further understand rural tourist behavior in the rural environment.

### Analysis the Spatial characteristic in rural landscape

To serve the tourist better, spatial morphology is the first step we should consider when planning and designing. At present, the development of rural tourism is not enough, but it is a hot spot for tourism. Spatial morphology is considered to be objective, relatively stable and quantitative due to its physical characteristics^36^. In the study of spatial morphology analysis, most of the research has predominantly focused on urban spatial morphology. As fine-grained data on land use and travel activities become available, they provide us with the opportunity to improve our understanding of the relationship between spatial morphology and transportation^37^. Based on the evaluation and discussion of the spatial morphology of urban areas, Jia identified a total of 17 indices of urban spatial morphology. By utilizing cluster analysis, Jia further divided the 146 cities into five clusters, highlighting significant regional variances among them^38^. Model imitation is another analytical method. In a study conducted by researcher Wu, it is argued that China’s urban growth can be classified into two predominant types: low-density expansion and high-density infill. The study suggests that these types might be influenced by many factors during different phases of development^39^. Multidisciplinary integration is a trend in the development of spatial morphological analysis. Landscape architects and researchers have merged spatial morphology with various disciplines, such as resilience science^40^, Social ecology^41^, environment science^42^, ‌transportation science^43^, climate science^44^, etc. In the research on rural spatial morphology, Agboola studied neighborhood-related challenges and investigated residents’ activity patterns within a rural neighborhood in southwestern Nigeria through morphological and GIS-based land use analysis^45^. From rural tourism perspective, Xi has summarized that the rural spatial morphology exhibits the village characteristics that correspond to three types of land development: “intensive reconstruction type,” “enclave extension type,” and “in situ utilization type,” respectively represented by “modern town,” “semi-urbanization,” and “traditional village”^46^.

### Exploring the Spatial characteristic in response to tourist behavior

It is vital to explore the relationship between tourist behavior and spatial characteristics. Rural tourism is still thriving, but the relationship hasn’t been fully understood and explored, which needs more effort put into this professional field. For a long time, researchers have studied tourist behavior in urban areas. In the city, the daily microclimate in each area affects the willingness of tourists to visit the city’s parks^47^. The researchers unveil some features of visitor behavior and spatial impact to shed light on the problem that tourists in museums often suffer from ‘hyper congestion’, which is key to improving the museum’s environment and visitor experience. Research on rural tourism is relatively limited, mainly focusing on some specific case studies. Ma has researched how human activities around the Miao villages interact with the natural environment and how that affects changes in the forest^48^. Through the application of space syntax theory, previous studies have investigated the relationship between human habitation and urban activity^49^. Using user-generated content (UGC) data from Mafengwo as a data source, spatio-temporal analysis techniques within GIS are employed to process and visualize the data in both time and space. This approach enables the analysis of the spatio-temporal characteristics and influencing factors of rural tourist behavior^50^. There are also numerous issues that need to be addressed, such as over-urbanized planning layout, ambiguous tourist destination signage, and a lack of regional characteristics. For rural tourism, which is distinctly different from city tourism, sustainable development is crucial^16^. However, space syntax has not been used to study the countryside, nor has it been integrated with tourist behavior. In terms of tourist data, it is relatively singular and weak, most studies only utilized traditional data sources (surveys and PRA) or only used one type of tourist data. Due to the complexity of the topography in rural areas, with mountains and buildings arranged in accordance with the terrain, pedestrian data is difficult to collect. This study utilized multisource data and employed a mixed-methods approach to obtain high-precision data, ensuring the accuracy of quantitative analysis. Meanwhile, tourists and the spatial environment were considered and analyzed separately after integration. For tourists, trajectory data and Weibo comments data were integrated. For spatial environment, passive layer and visual layer were considered separately. Understanding people’s views on rural tourism is crucial for the effective management and planning of rural landscapes^51^.

### Research objective

Surveys in rural areas are both time-consuming and labor-intensive, and there are issues with data incompleteness. The problem with big data mainly lies in the fact that many people do not have technical equipment, leading to the incompleteness of current rural spatial morphology data and difficulties in statistical analysis of rural tourist behavior data. Literature on spatial characteristics has mainly focused on urban spaces. Additionally, rural spatial morphology studies mainly focus on the classification of spatial types, with most adopting a single-dimensional research approach, rarely paying attention to the tourists’ behavior.

There is limited research on the spatial morphology related to rural landscapes to inform the tourists’ behavior for the rural tourism. Therefore, this study combines multi-source tourist data, including tourist trajectory data and tourists’ Weibo comments, as well as various technological methods to address the research question of exploring relationship between tourist behavior and rural spatial characteristic. The main research objectives of this study are as follows:

1. expand the research setting from the urban to the rural.
2. better understand tourist behavior by innovating the rural research method and using more efficient research and assessment methods.
3. better realize rural tourism planning through studying tourist behavior.

This study aims to provide suggestions for optimizing future rural tourism planning and guides for the overall planning of rural tourism.

## **Methodology**

### Study area

It is an outstanding representative of Huizhou culture, Hui-style architecture, and the scenery of southern Anhui (Fig. 2a)^52,53^. Hongcun Village is rich in tourism resources, recognized as a UNESCO World Heritage Site for its well-preserved traditional Anhui architecture and planning^54^. The village has 137 buildings, which were constructed during the Ming and Qing dynasties, showcasing the distinctive features of Hui-style architecture, including whitewashed walls, black tiles, and intricately carved wooden structures^55^. The unique landscape of the traditional village in southern Anhui province is constituted by the unique cattle horn-shaped village structure, the artificial ancient water system, the excellent architectural artistry, and the picturesque pastoral landscape (Fig. 2b). The village possesses significant historical, artistic, and scientific value. It serves as the living fossil of Huizhou culture and is known as a “village in traditional Chinese painting”^56^. Hongcun Village was inscribed on the UNESCO World Cultural Heritage List in 2000 and was rated as a 5 A scenic spot in China in 2011^57^.

### Study strategies

**Fig. 1:**
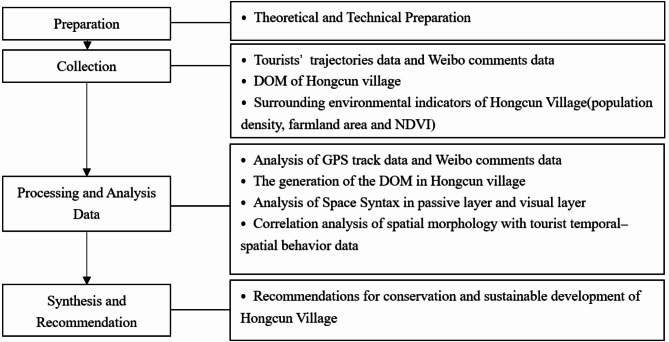
Study stages.

Presently, most studies only utilized traditional data sources (surveys and PRA)^19^ or only used one type of tourist data^58^. This leads to the data in rural studies being singular and weak. This study applied a mixed-methods strategy utilizing multisource data to achieve high-precision data, hence insuring the correctness of quantitative analysis. Tourists and the spatial environment were analyzed and considered independently following their integration. For tourists, we respectively obtained trajectory data and Weibo comments data using Python from two outdoor tourist websites (www.foooooot.com and www.2bulu.com) and Weibo (www.weibo.com)^59^. For spatial environment, while considering the internal environment of Hongcun Village, the surrounding environment of Hongcun Village was also considered. For the interior of Hongcun Village, DOM captured by UAV was used, combined with spatial syntax, and the passive layer and visual layer were considered separately. For the surrounding environment of Hongcun Village, we divided the surrounding area into four zones and obtained and analyzed the farmland area, surrounding town population density, and NDVI for these four zones, corresponding them one by one with the internal space of Hongcun Village. These methods and technologies are integrated to address the research question of exploring the relationship between tourist behavior and rural spatial characteristic. The stages of the research include preparation, collection, processing and analysis data, also synthesis and recommendation (Fig. 1).

### Data collection

#### Acquisition of trajectories data and Weibo comments data

This study takes the tourists who visit the core heritage area of Hongcun Village as the study subjects.

However, due to the impact of the COVID-19 pandemic, traditional data collection methods such as questionnaires and handheld GPS data loggers are difficult to implement efficiently. Therefore, the study employs Python scripts to collect all the tourists’ trajectories data, which is voluntarily uploaded by tourists on the websites www.foooooot.com and www.2bulu.com from 2011 to 2024. These two platforms have their own tracking apps, which are installed on mobile devices and are widely used in China. The trajectory data obtained from these apps provides touring information, including start and end times, average speed, travel length, total travel time and overall satisfaction. For obtaining Weibo comments data, Python scripts are also used to collect Weibo comments data on the website www.weibo.com between 2023 and 2024, including comment content and uploaded images.

#### Acquisition of UAV remote sensing data

UAVs have been widely used in urban research, but rarely used in rural research^60^. The primary reason is the complexity of rural landscapes, which makes it difficult for UAVs to plan routes for extensive aerial photography and flight study, unlike in metropolitan areas. UAVs typically need to fly manually in order to navigate complex terrain.

**Fig. 2:**
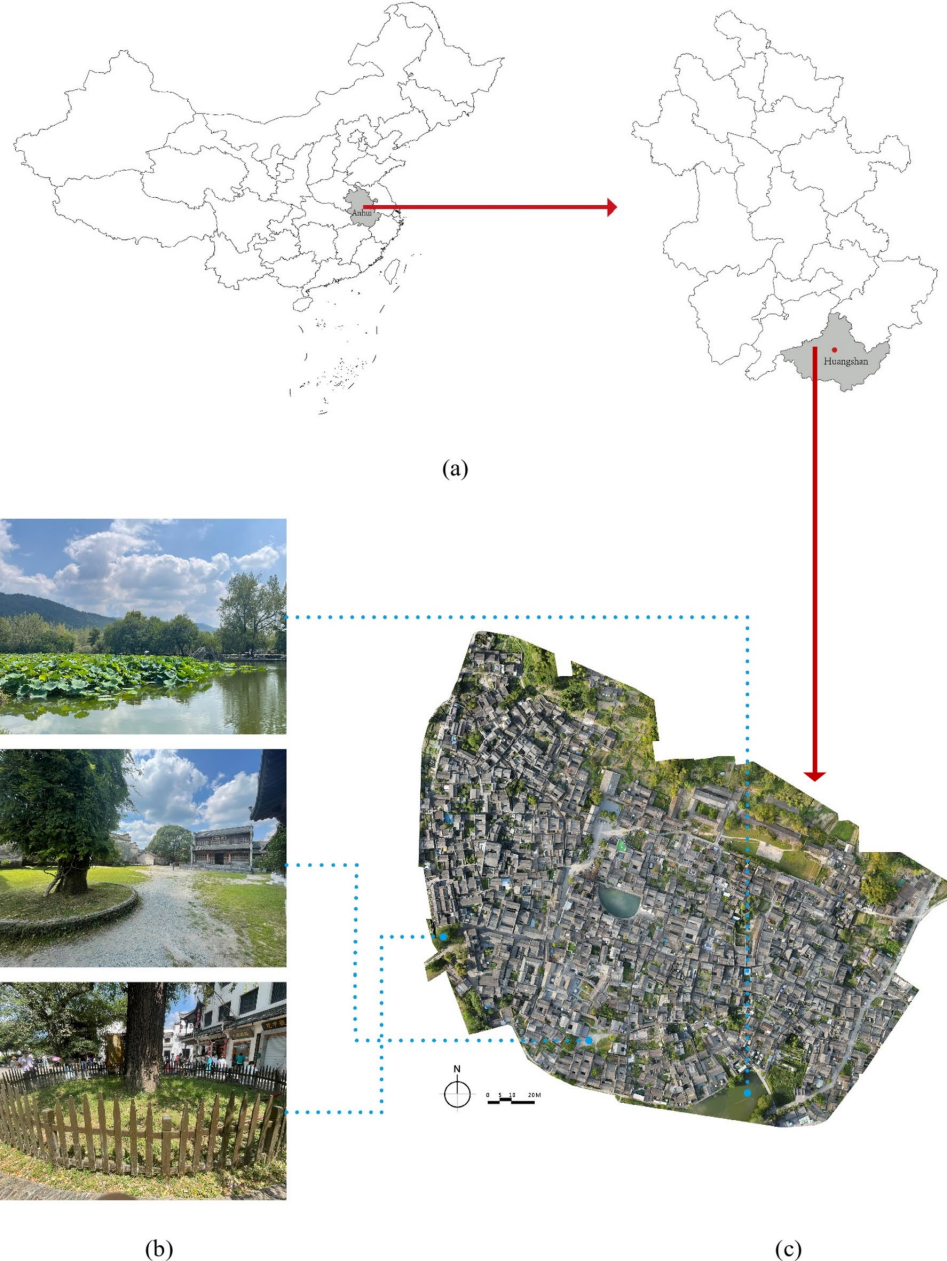
Hongcun Village. (**a**) the location of the study area in China and the location of Hongcun Village in Anhui Province; (**b**) the photographs of Hongcum village; (c) the DOM of Hongcun Village. Figure was created using Pix4Dmapper 4.4.12 (https://www.pix4d.com/).

UAV (Unmanned Aerial Vehicle) is utilized to gather remote sensing data since this work needs a high-precision DOM (Digital Orthophoto Map) to examine the spatial syntax of Hongcun village. For better resolution and cartographic accuracy, the FPV (First Person View) tilt angle was fixed to 90° when the UAV was utilized at a low altitude, 70 m above the ground, in this investigation. The weather on the day of the survey was suitable for the UAV’s remote sensing needs: there was enough sunshine, the wind speed of 10 km/h, and no electromagnetic interference in the immediate area. The flight speed is 5 m/s, and the interval of aerial photography is 5 s. In order to guarantee that the horizontal and vertical pixels of every image overlap more than one-third, the study was taken in a “zigzag” pattern. The DOM of Hongcun village was created by using the Pix4Dmapper 4.4.12. Figure 2c illustrates the DOM that is the result of UAV remote sensing data integration.

### Data analysis method

#### GPS track data analysis

GPS Track Data records the location data generated by tourists in the process of travel, allowing for a direct visualization of their travel track. Through effective utilization of this data, it can reveal other specific spatio-temporal behaviors of tourists^61^. There are 4457 trajectories data on the two outdoor platforms from 2011 to 2024 with Hongcun Village as the key word. Through preliminary screening, there are 2661 trajectories data located in the core heritage area of Hongcun Village from 2011 to 2024. However, because of the interference from cell phone signals and other circumstances, the distribution of certain trajectories is scattered, lost or disconnected. By removing these useless trajectories, a total of 1380 trajectories were available used for further study analysis. This dataset comprised 358,491 valid GPS points, resulting in a validity rating of 76.4%.

Each trajectory was downloaded from the two platforms in the form of a .GPX file, which contains essential information such as datetime and geographical coordinates. The first step was to import the trajectory data into ArcGIS, excluding the GPS points that were outside the study area, and converting them into .XLS files. In the second step, Pandas was utilized to calculate the time, the distance, and the average speed between two adjacent points. The formula is as follows:

$$\:{D}_{i}=R\times\:{{cos}}^{-1}\left[{cos}{y}_{i}{cos}{y}_{m}{cos}\left({x}_{i}-{x}_{m}\right)+{sin}{y}_{i}{sin}{y}_{m}\right]$$

$$\:m=i-1$$

$$\:{AS}_{i}=\frac{{D}_{i}}{{DT}_{i}}$$

$\:{D}_{i}$ is the distance between GPS point $\:i$ and point $\:m$, where $\:i\:\text{r}\text{a}\text{n}\text{g}\text{e}\text{s}\:\text{f}\text{r}\text{o}\text{m}\:2\:\text{t}\text{o}\:n\:$($\:n$ represents the total number of GPS points on the same track). $\:R$ indicates the value of the radius of the earth, $\:{x}_{i}$ is the longitude coordinate of point $\:i$, $\:{y}_{i}$ is the latitude coordinate of point $\:i$, $\:{AS}_{i}$ represents average speed value from GPS point $\:m$ to point $\:i$, and $\:{DT}_{i}\:$denotes the time spent between GPS point $\:i$ and point $\:m$.

In the third step, we filtered out invalid GPS data points that had a time gap exceeding 60 s between them. Additionally, if the distance between two neighboring locations exceeded 50 m, it was deemed unnatural and subsequently removed. Considering that pedestrian speed is generally about 1.5 m/s, so any average speed exceeding this threshold was also deleted. In the fourth step, we removed the points that took little time but were far apart away from another point. The rules for cleaning data are shown in Table 1.

**Table 1:** The rules for cleaning GPS initial data.

Num	Variable	Description of cleaning rules
1	Point	If the time of the adjacent GPS track point were more than 60s
2	Distance	If the distance of the adjacent GPS track point exceeded 50 m
3	Speed	If the speed of GPS track point was greater than 1.5 m/s or less than 0 m/s
4	Position	The position of the adjacent GPS track point was far away

The fifth step was to calculate the dwell time, distance and speed based on 1380 trajectories data. Table 2 shows an example of GPS point data. The attribute information for each GPS point includes the trajectory number, the sequence number of the point within the trajectory, timestamp information, longitude, latitude, dwell time spent, the distance between two adjacent points and speed.

**Table 2:** GPS data attribute values.

Name	Number	Original number	Datetime	Longitude	Latitude	Dwell time (s)	Distance (m)	Speed (m/s)
1	9	10	2018-07-11 07:08:25	117.9866	30.00454	29	5.291	0.182
1	10	12	2018-07-11 07:09:37	117.9865	30.00463	5	5.237	1.047
1	11	14	2018-07-11 07:12:23	117.9863	30.00458	40	8.295	0.207
1	12	15	2018-07-11 07:12:35	117.9863	30.00458	12	1.908	0.159
1	13	16	2018-07-11 07:12:58	117.9863	30.00453	23	6.119	0.266
1	14	17	2018-07-11 07:13:10	117.9862	30.00448	12	5.415	0.451
1	15	18	2018-07-11 07:13:30	117.9863	30.00456	20	6.569	0.328
1	16	19	2018-07-11 07:13:37	117.9863	30.00452	7	4.085	0.584
1	17	20	2018-07-11 07:13:46	117.9862	30.00444	9	6.592	0.732
1	18	21	2018-07-11 07:13:54	117.9861	30.00433	8	9.43	1.179
1	19	23	2018-07-11 07:14:08	117.986	30.00419	8	4.491	0.561

**Fig. 3:**
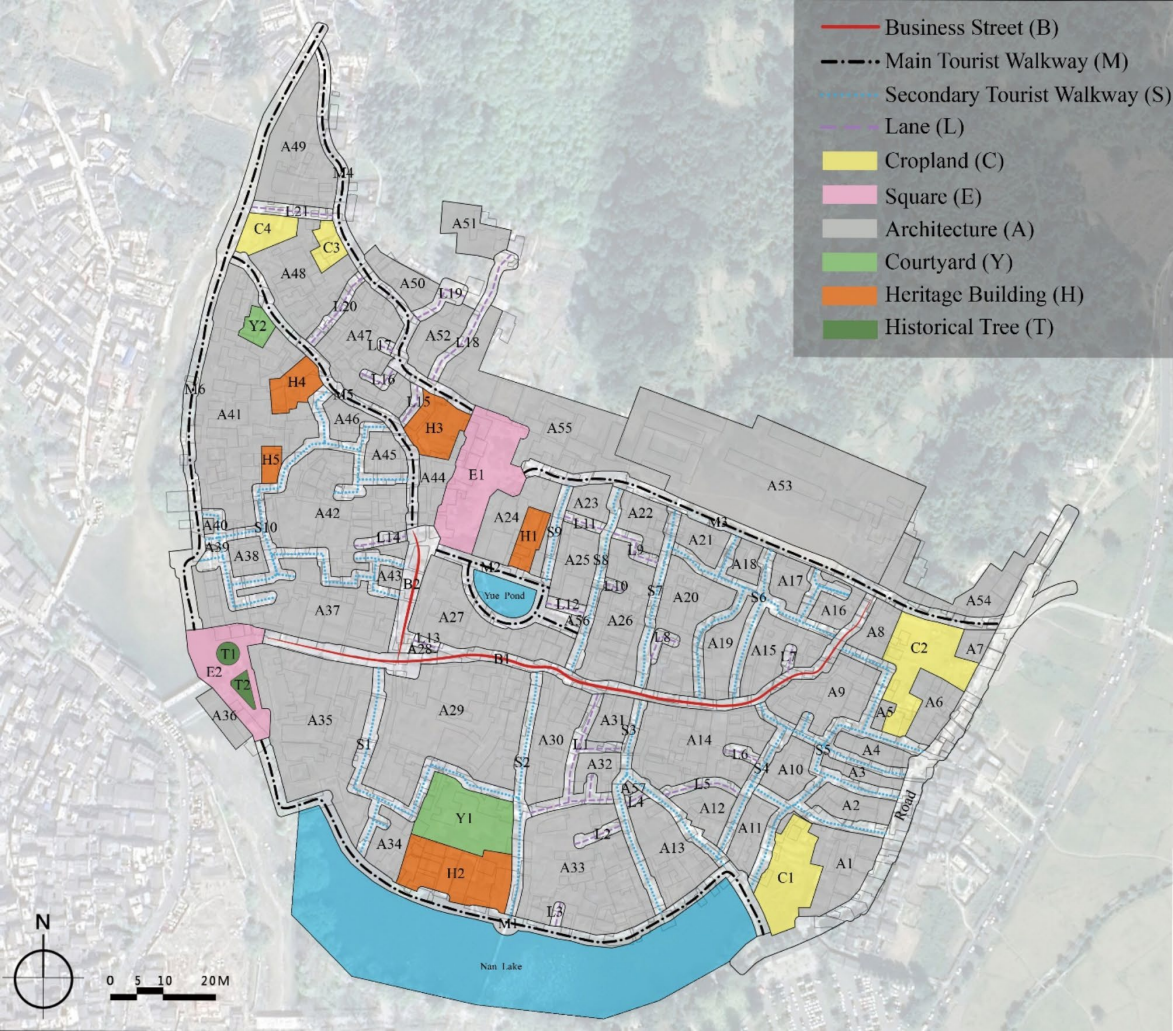
Different types of spaces of Hongcun Village. Figure was created using ArcGIS PRO 3.0(https://www.esri.com/en-us/home).

Since the village is open and has multiple entrances, researchers are unable to control the starting and ending points of the GPS track. It is unreasonable to reflect the actual characteristics of tourist behavior by using the whole tour time and track length. Therefore, as depicted in Fig. 3, all the elements in the village have been split into 109 spaces and labeled according to various space qualities, including forest spaces, building spaces, open spaces, and pedestrian spaces.

In the sixth step, four indicators were employed to indicate the spatio-temporal behaviors of tourists in different spaces: Visiting Proportion, Average Dwell Time, Average Distance and Average Speed. ‘Visiting Proportion’ shows the number of tourists who visited a specific space as a percentage of the total number of tourist groups. ‘Average Dwell Time’ means that the average duration of time that tourists spend in the space. ‘Average Distance’ means that the average value of distance tourists move in the particular space. ‘Average Speed’ means tourists’ speed in a specific space. These four indicators can accurately reflect the popularity of the specific space for tourists. For each space, the $\:Visiting\:Proportion$, $\:Average\:Dwell\:Time$, $\:Distance$ and $\:Average\:Speed$ were calculated as follows:

$$\:Visiting\:Proportion=\frac{Number\:of\:visitors\:}{Total\:number\:of\:visitors}\times\:100\%$$

$$\:Average\:Dwell\:Time=\frac{Sum\:of\:visito{r}^{{\prime\:}}s\:dwell\:time\:}{Number\:of\:visitors\:}$$

$$\:Average\:Distance=\frac{Sum\:of\:travel\:distance}{Number\:of\:visitors\:}$$

$$\:Average\:Speed=\frac{Sum\:of\:visitors{\prime\:}\:speed}{Number\:of\:GPS\:points}$$

**Fig. 4:**
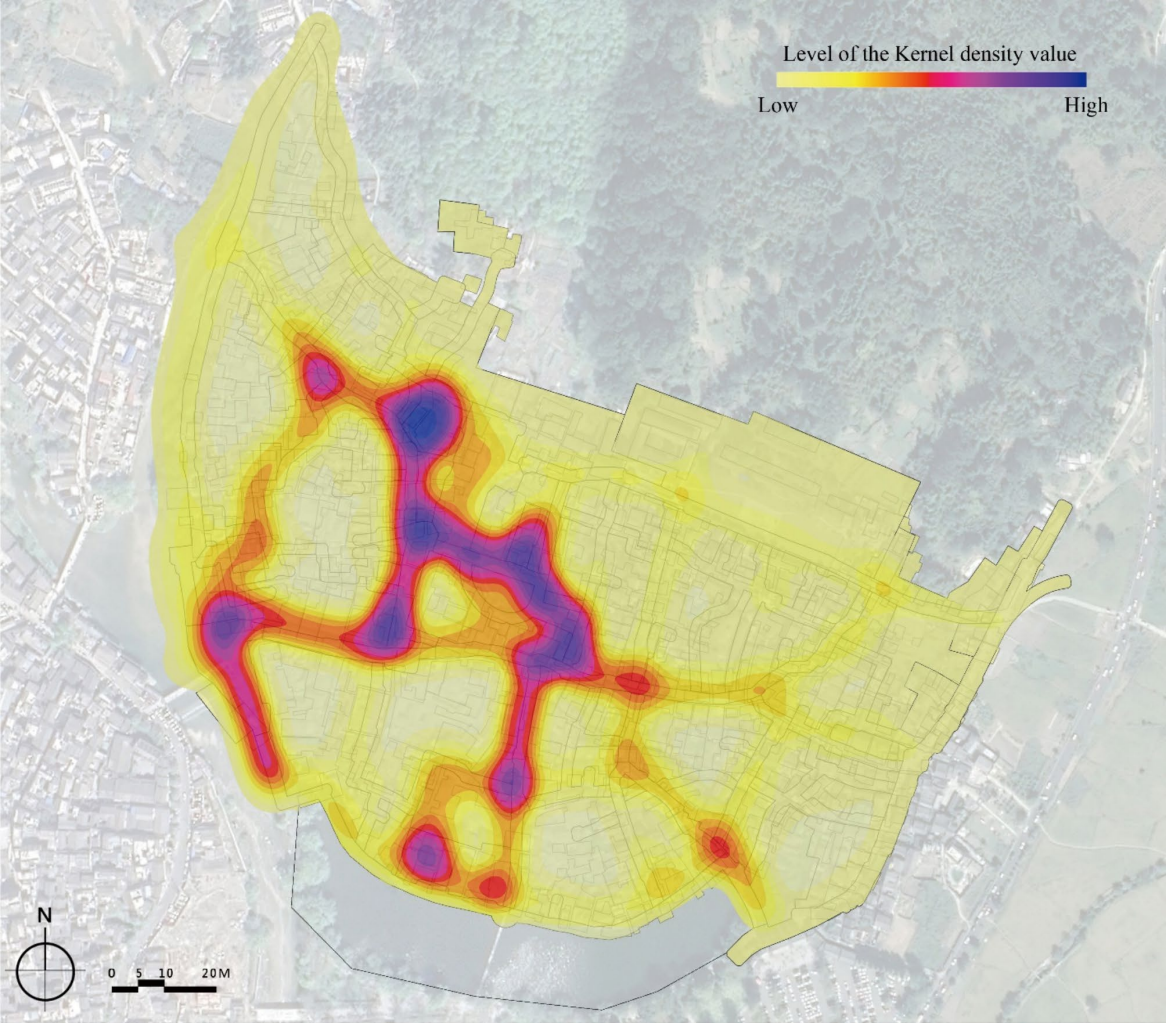
The Kernel density analysis result for 1380 visitor trajectories. Figure was created using ArcGIS PRO 3.0(https://www.esri.com/en-us/home).

The seventh step was to merge the 1380 trajectories into one dataset using ArcGIS PRO 3.0 and the Kernel density analysis tool. The result was produced using a search radius of 25 m and the output raster cell size is 5 × 5 m^2^, as shown in Fig. 4.

#### Weibo comments data

Nowadays, more and more research uses social media sources to analyze spatiotemporal behavior, including geotagged photos on Flickr^62^, Facebook posts^63^, and Weibo check-ins^64^. Weibo, as one of the most popular online social media and social networking platforms in China, has been used in some studies to analyze the spatiotemporal behavior of Chinese tourists in mainland Chinese cities^65^.

This study uses Python to collect Weibo comments data related to “Hongcun.” We set the keyword as “Hongcun” and collected all Weibo posts containing images from January 2023 to November 2024. There are 21,459 Weibo comments data and photos tagged with the location of Hongcun village. The content includes comment text, posting time, posting IP, number of likes, and basic user information, ensuring the data source is legal and compliant. The text data processing steps are as follows:

1. Preliminary screening and processing: Filter the obtained data to remove meaningless, duplicate, and content-mismatched invalid content, ensuring data quality.
2. Keyword filtering: Use Excel’s filtering function to filter blog posts by keywords, selecting 46 terms such as Chengzhi Hall, Wang Family Ancestral Hall, Green Garden, the alley, and Qian Street, and count the frequency of each keyword.
3. Regional positioning: Assign the 46 filtered keywords to 109 regions, with each region corresponding to 1–2 keywords.

#### Space syntax and syntactic variables

Initially, space syntax research focused on pedestrian mobility in cities^66^, but later, space syntax research evolved into much more interdisciplinary work^67^. Human behavior has been found to be highly associated with several topological measurements of space syntax in studies^68^. Tourist behavior is a kind of the human behavior. As a result, we believe that space syntax may be utilized to evaluate tourist behavior, as it provides an explanation of the link between human activity in space and spatial morphology^67^. Space syntax offers a new perspective for examining urban environments from physical, morphological, and social angles. This research uses space syntax as a reference for exploring the traditional village spaces.

Depthmap X is a multi-platform software platform to perform a set of spatial network analyses designed to understand social processes within the built environment. It works at a variety of scales from building through small urban to whole cities. At each scale, the aim of the software is to produce a map of open space elements, connect them via some relationship (for example, intervisibility or overlap), and then perform a graph analysis of the resulting network^69^. Space syntax software provides the necessary graphic aid in understanding the current situation of designed spaces with respect to accessibility and visibility^70^.

Space syntax analysis of Hongcun was conducted from the Passable Layer and the Visible Layer through Depthmap. The former refers to all spaces accessible to tourists on foot, excluding rural water bodies, farmlands, and impassable green spaces. The latter means the full range of spaces that are visible to tourists as they walk through rural streets, alleys, and open public spaces.

First, use AutoCAD to trace the road and building layout of Hongcun based on the UAV-captured DOM, generating a .dxf file. Then, import the .dxf file into Depthmap X to conduct graph analysis in the Passable Layer and visibility graph analysis in the Visible Layer, performing topological calculations with the software. The village is dominated by Hui-style ancient dwellings, which feature courtyards and high protective walls^71^, known locally as horse-head walls, fencing the facades of the buildings. Therefore, tourists can’t see the interior of the buildings when they walk around. In this way, not only is the privacy of local rural residents protected, but the characteristics of Huizhou culture are fully exposed to tourists. Therefore, the interior space of the building is excluded from the Visible Layer. For the above reasons, the edge length of Depthmap’s grid was adjusted to 1000 mm based on the narrowest lanes and walkway widths, as well as the human size.

Connectivity, Control, Mean Depth, Integration, and Intelligibility were selected as common syntactic variables for assessing the diverse spatial characteristics of Hongcun Village in Depthmap under both the Passable Layer and the Visible Layer. The Visual Clustering Coefficient was a syntactic variable for the Visible Layer. The specific variables and their descriptions are summarized in Table 3.

**Table 3:** Space evaluation indicators used in this study.

Variable	Description	Value
Connectivity	The higher the value, the more the space connects with others	Maximum value
Control	The higher the value, the more significant the space in the system	Maximum value
Mean depth	The lower the value, the better the spatial accessibility	Minimum value
Integration	The higher the value, the more convenient the space	Maximum value
Visual clustering coefficient	The higher the value, the more restrictive eyesight line will be affected by space boundary	Minimum value
Intelligibility	The higher the value, the higher the visual permeability of the space	Connectivity/Integration

##### Connectivity

Connectivity is clearly a property that can be seen from each space, where one in the space can see how many neighboring spaces it connects to^67^. A higher Connectivity value indicates a greater connection between one space and others. The formula is as follows:

$$\:{Con}_{k}=h$$

In the given equation, $\:{Con}_{k}$ represents the connectivity value of space $\:k$, where h indicates the number of spaces connected to space $\:k$. Here, $\:k\:ranges\:from\:1\:to\:n$ ( $\:n$ represents the total number of spaces in the study area).

##### Control

The control value describes how space $\:k$ controls some other spaces related to it^72^. The higher the control value, the less spaces are controlled by space $\:k$. In other words, a higher control value indicates the greater significance of space $\:k$ in the system. The visual control value denotes how the viewpoint $\:g$ controls some other viewpoint that can be seen by it in the system. The formula is as follows:

$$\:{Ctr}_{k}={\sum\:}_{l=1}^{m}\:1/{Con}_{l}$$

$$\:{VC}_{g}=\frac{{\sum\:}_{f=1}^{{N}_{g}}{Ctr}_{f}}{{Ctr}_{g}}$$

In the first given equation, $\:{Ctr}_{k}$ is the control value of space $\:k$, where space $\:l$ represents the spaces connected with space $\:k$. And $\:{Con}_{l}$ refers to the number of spaces connected with space $\:l$, where $\:\:l=\text{1,2},\text{3,4},\dots\:,\:m$. In the second given equation, $\:{VC}_{g}$ represents the visual control value of viewpoint $\:g$. And $\:{Ctr}_{f}$ denotes the control value of each viewpoint that can be seen in the sight of viewpoint $\:g$, where $\:f=\text{1,2},\text{3,4},\dots\:,\:{N}_{g}$. $\:{N}_{g}$ indicates the number of viewpoints that can be seen by viewpoint $\:g$, and $\:{Ctr}_{g}$ stands for the control value of viewpoint $\:g$.

##### Mean depth

The depth value denotes distance from one space to another space in the system. Distance is defined as the fewest number of nodes required to connect two spaces^67^.

The total depth value refers to the sum of distances from space $\:k$ to each space else in the system. The mean depth value is the average depth from space $\:k$ to all the other spaces^67^. The formula is as follows:

$$\:{MD}_{k}=\frac{{TD}_{k}}{{N}_{a}-1}=\frac{{\sum\:}_{l=1}^{{N}_{a}}{D}_{kl}}{{N}_{a}-1}$$

In the given equation, $\:{MD}_{k}$ refers to the mean depth value of space $\:k$, $\:{TD}_{k}$ represents the total depth value of space $\:k$, $\:{N}_{a}$ is the sum of spaces in the study, and $\:{D}_{kl}$ indicates the fewest distance from space $\:k$ to space $\:l$.

##### Integration

The integration value is an element within a complex that can evaluate the accessibility between space $\:k$ and all other spaces in the system. It is not visible from a space since it sums up the depth of the space from all others, the majority of which are not visible from that space^67^. Relativized RA(RRA) is the reciprocal of integration. Relativized Asymmetry (RA) can eliminate the influence of topology asymmetry. The formula is as follows:

$$\:{Ita}_{k}=\frac{1}{{RRA}_{k}}=\frac{1}{\frac{{RA}_{k}}{{RA}_{D}}}=\frac{{RA}_{D}}{\frac{2\left({MD}_{k}-1\right)}{{N}_{a}-2}}$$

$$\:{RA}_{D}=\frac{2\left\{N\left[{\text{log}}_{2}\frac{N}{3}-1\right]+1\right\}}{\left(N-1\right)\times\:\left(N-2\right)}$$

In the given equation, $\:{RA}_{D}$ refers to the RA of diamond-shaped topology which is the normalization formula of RA^73^. $\:{RRA}_{k}$ stands for RRA value of space $\:k$, $\:{MD}_{k}$ is the mean depth value of space $\:k$, $\:{N}_{a}$ represents the sum of spaces in the system, and $\:N$ indicates the number of elements in diamond-shaped topology.

##### Intelligibility

The attribute of ‘intelligibility’ refers the extent to which we can perceive from the spaces that constitute the system. An intelligible system has well-connected and well-integrated spaces^67^. Therefore, the intelligibility value is related to the integration value and connectivity.

##### Visual clustering coefficient

The visual clustering coefficient value refers to the limitation of eyesight line that influenced by space boundary^74^. The higher the visual clustering coefficient value, the more restrictive eyesight line will be affected by space boundary. The formula is as follows:

$$\:{VCC}_{g}=\frac{{K}_{g}}{k\left(k-1\right)}$$

In the given equation, $\:{VCC}_{g}$ refers to the visual clustering coefficient value of viewpoint $\:g$, $\:{K}_{g}$ denotes the number of elements visible from viewpoints which are in the sight of viewpoint $\:g$, excluding $\:k$, and $\:k$ indicates the number of elements can be seen by viewpoint $\:g$.

#### Surrounding environment data

The remote sensing data used in this paper comes from the 1 m resolution land cover map of China in the Zenodo data management system. This dataset, based on deep learning frameworks and open-access data (including the Global Land Cover (GLC) products, OpenStreetMap (OSM), and Google Earth images), has established China’s first national-scale land cover map at 1 m resolution, SinoLC-1^75^. Select the land cover map of Huangshan City, Anhui Province, and perform NDVI calculations for the four districts separately. Then, use the pure pixel method to invert the forest vegetation coverage, ultimately obtaining the vegetation coverage for the four districts.

$$\:\varvec{F}\varvec{V}\varvec{C}=\frac{(\varvec{N}\varvec{D}\varvec{V}\varvec{I}-{\varvec{N}\varvec{D}\varvec{V}\varvec{I}}_{\varvec{s}\varvec{o}\varvec{i}\varvec{l}})}{({\varvec{N}\varvec{D}\varvec{V}\varvec{I}}_{\varvec{v}\varvec{e}\varvec{g}}-{\varvec{N}\varvec{D}\varvec{V}\varvec{I}}_{\varvec{s}\varvec{o}\varvec{i}\varvec{l}})}$$

In the given equation, $\:FVC$ refers to Fraction of Vegetation Cover, $\:{\varvec{N}\varvec{D}\varvec{V}\varvec{I}}_{\varvec{s}\varvec{o}\varvec{i}\varvec{l}}$ denotes the NDVI value of soil index, $\:{\varvec{N}\varvec{D}\varvec{V}\varvec{I}}_{\varvec{v}\varvec{e}\varvec{g}}$ indicates the NDVI value of vegetation index.

The farmland area refers to the land area within a certain geographical range used for planting crops, including arable land, terraced fields, and garden fields. It reflects land use patterns, agricultural development levels, and ecological environment conditions. This paper uses the 1 m resolution land cover map of Huangshan, employing image processing technology to extract farmland areas, thereby calculating the farmland coverage areas corresponding to the four districts.

The population data in this article is sourced from WorldPop, the spatial distribution of population in 2020, China^76^. This article uses the 100 m resolution population density map of Huangshan, processed through ArcGIS PRO 3.0, to calculate the population density of the corresponding areas in the four districts.

## Results

### Data analysis results

#### GPS track data

Table 4 shows the pertinent example attribute values for different spaces in Hongcun village.

**Table 4:** Example attribute values for different spaces.

Space name	Visiting proportion (%)	Average dwell time (min)	Average distance (m)	Average speed (m/s)
A1	7.02	0.71	27.01	0.82
A2	5.79	0.74	21.43	0.75
B1	91.27	5.59	186.25	0.81
B2	81.56	2.52	68.50	0.60
C1	7.28	0.35	10.28	0.68
C2	4.58	0.32	20.88	1.12
H1	51.33	1.88	38.98	0.43
H2	61.27	2.45	58.60	0.57
L1	36.52	1.34	42.66	0.69
L2	1.80	0.56	14.20	0.81
M1	91.55	4.16	142.13	0.69
M2	85.43	5.50	150.13	0.52
E1	53.58	2.45	76.61	0.65
E2	83.15	3.08	88.03	0.59
S1	35.73	1.22	40.32	0.77
S2	69.91	2.58	65.49	0.58
Y1	53.52	2.88	71.11	0.62
Y2	5.33	0.62	18.41	0.66

**Fig. 5:**
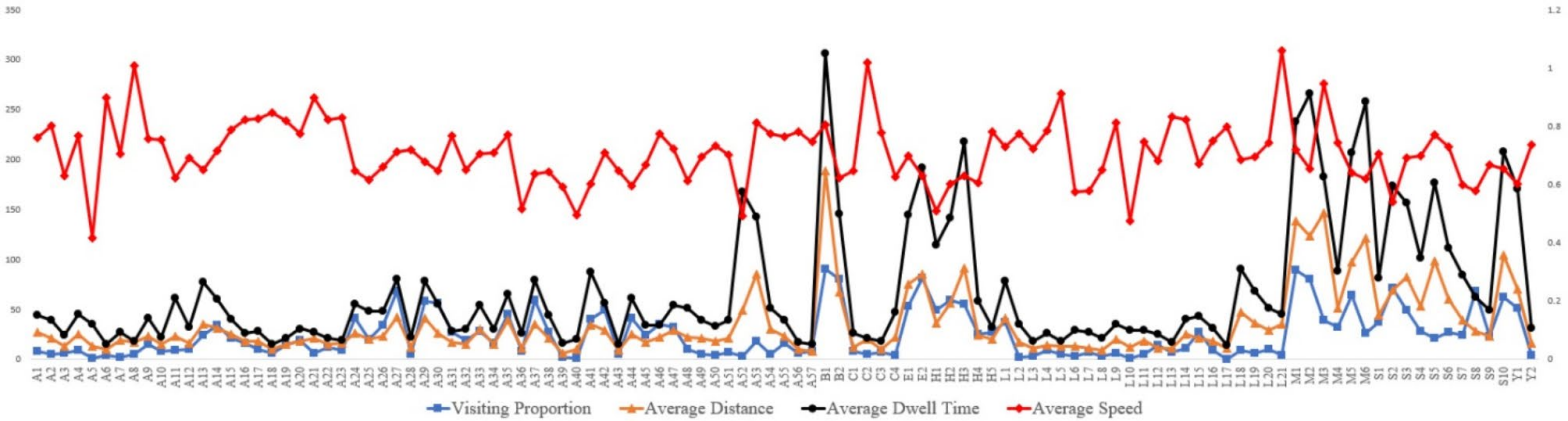
depicts a line chart illustrating the visitor behavior data from the 1380 trajectories in 109 spaces.

Figure 5. The average values of Visiting Proportion, Average Dwell Time, Average Distance, and Average Speed for visitor trajectories in 109 spaces.

#### Space syntax

**Fig. 6:**
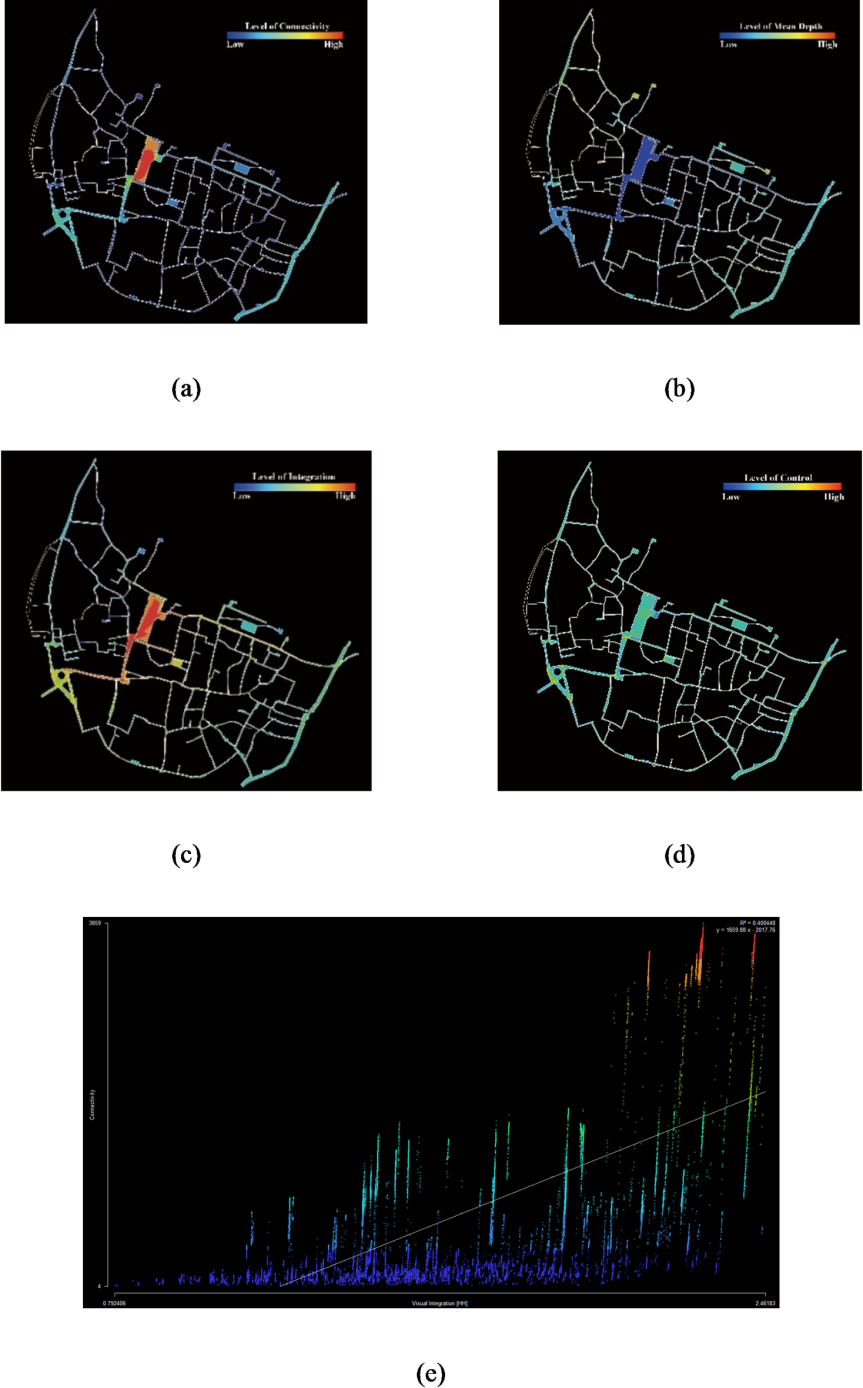
Depthmap analysis results for Hongcun village in Passable Layer. (**a**) Connectivity; (**b**) Mean Depth; (**c**) Integration; (**d**) Control; (**e**) Intelligibility. Figure was created using Depthmap X (https://www.spacesyntax.online/).

**Fig. 7:**
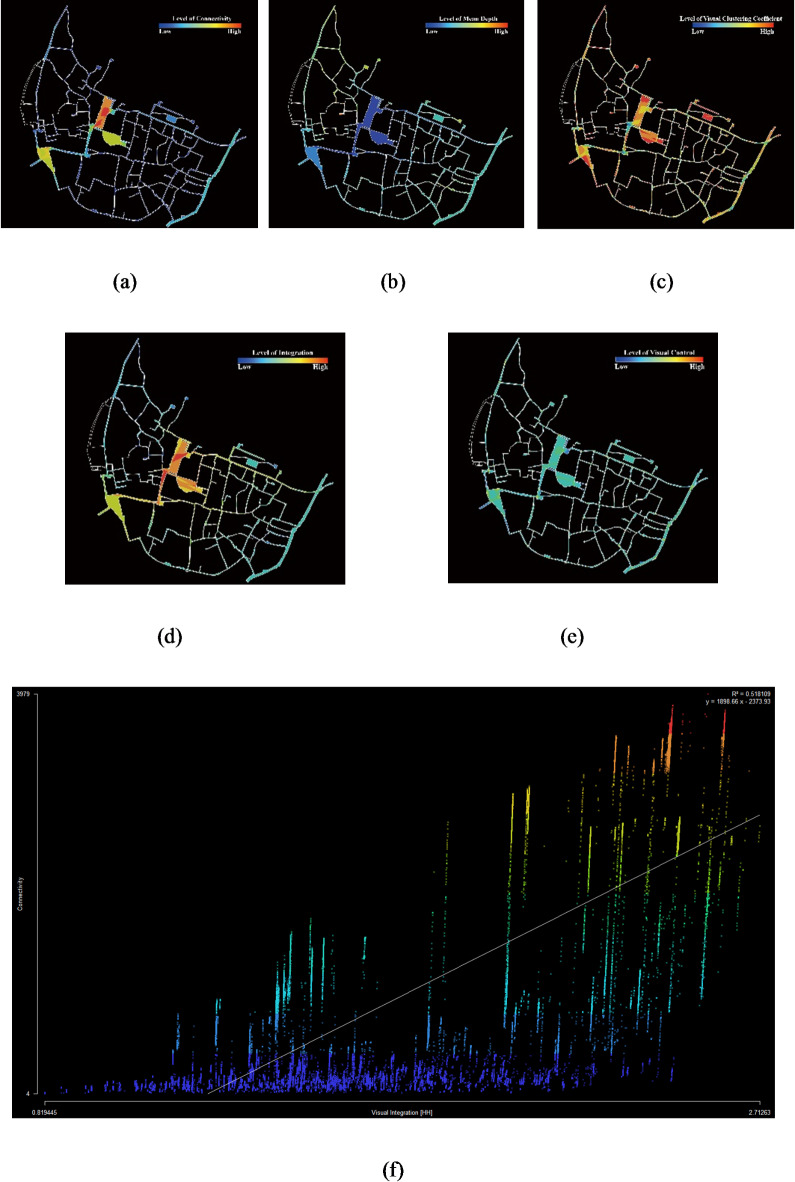
Depthmap analysis results for Hongcun village in Visible Layer. (**a**) Connectivity; (**b**) Mean Depth; (**c**) Visual Clustering Coefficient; (**d**) Integration; (**e**) Control; (**f**) Intelligibility. Figure was created using Depthmap X (https://www.spacesyntax.online/).

Figures 6 and 7 show the findings of the analysis conducted on the Visible Layer and the Passable Layer generated from Depthmap, where the colors in the Intelligibility map indicate the spaces with the same color in the Integration map.

### Correlation analysis of Spatial morphology with tourist temporal–spatial behavior data

In this study, there are a total of 109 spaces in Hongcun, comprising 68 architectural spaces and 41 outdoor public spaces. Since the architectural spaces in the village are relatively independent, the space syntax analysis can only be conducted on the 41 public areas and spaces visible to tourists.

Based on variance analysis and a normal distribution test of Visiting Proportion, Average Dwell Time, Average Distance, Average Speed, Weibo comments, population density, NDVI and farmland area, and the results of space syntax analysis of various spaces through IBM SPSS Statistics 26.0, it was observed that the data samples in this study are not normally distributed. As a consequence, for the correlation analysis, the non-parameter Spearman analysis was applied. Tables 5 and 6, respectively, exhibit the findings for the Passable Layer and the Visible Layer.

**Table 5:** Correlation analysis between tourist behavior and Spatial environment in the passable layer.

		Connectivity	Control	Mean Depth	Integration	Intelligibility	Population density	NDVI	Farmland area
N		41	41	41	41	41	41	41	41
Visiting proportion	The correlation coefficient	0.685**	0.605**	− 0.378*	0.399**	0.683**	0.149	− 0.363**	− 0.189*
	Sig. (2 tails)	0.000	0.000	0.015	0.010	0.000	0.123	0.000	0.049
Average dwell time	The correlation coefficient	0.705**	0.643**	− 0.097	0.128	0.789**	0.234*	− 0.372**	− 0.319**
	Sig. (2 tails)	0.000	0.000	0.547	0.427	0.000	0.014	0.000	0.001
Average Distance	The correlation coefficient	0.728**	0.653**	− 0.095	0.125	0.821**	0.236*	− 0.353**	− 0.327**
	Sig. (2 tails)	0.000	0.000	0.555	0.436	0.000	0.014	0.000	0.001
Average speed	The correlation coefficient	− 0.036	− 0.063	0.027	− 0.035	− 0.027	− 0.194*	0.247**	0.082
	Sig. (2 tails)	0.822	0.695	0.868	0.828	0.869	0.043	0.010	0.398
Weibo COMMENTS	The correlation coefficient	0.433**	0.157	− 0.331*	0.337*	0.382*	− 0.121	0.029	− 0.080
	Sig. (2 tails)	0.005	0.327	0.035	0.031	0.014	0.210	0.763	0.409

**At level 0.01 (2 tails), the correlation was significant.

*At level 0.05 (2 tails), the correlation was significant.

**Table 6:** Correlation analysis between tourist behavior and Spatial environment in the visible layer.

		Connectivity	Visual clustering coefficient	Control	Mean Depth	Integration	Intelligibility	Population density	NDVI	Farmland area
N		41	41	41	41	41	41	41	41	41
Visiting proportion	The correlation coefficient	0.664**	− 0.605**	0.484**	− 0.382*	0.405**	0.679**	0.156	− 0.363**	− 0.190*
	Sig. (2 tails)	0.000	0.000	0.001	0.014	0.009	0.000	0.123	0.000	0.048
Average dwell time	The correlation coefficient	0.626**	− 0.493**	0.594**	− 0.088	0.114	0.721**	0.234*	− 0.372**	− 0.314**
	Sig. (2 tails)	0.000	0.001	0.000	0.586	0.477	0.000	0.014	0.000	0.001
Average Distance	The correlation coefficient	0.643**	− 0.497**	0.616**	− 0.083	0.107	0.736**	0.236*	− 0.353**	− 0.321**
	Sig. (2 tails)	0.000	0.001	0.000	0.608	0.507	0.000	0.014	0.000	0.001
Average speed	The correlation coefficient	− 0.151	0.086	− 0.064	0.021	− 0.029	− 0.037	− 0.194*	0.247**	0.085
	Sig. (2 tails)	0.713	0.595	0.691	0.896	0.859	0.820	0.043	0.010	0.381
Weibo comments	The correlation coefficient	0.496**	− 0.006	0.110	− 0.341*	0.348*	0.492**	− 0.121	0.029	− 0.082
	Sig. (2 tails)	0.001	0.968	0.492	0.029	0.026	0.001	0.210	0.763	0.399

**At level 0.01 (2 tails), the correlation was significant.

*At level 0.05 (2 tails), the correlation was significant.

### Tourist temporal–spatial behavior characteristics of rural tourism in Hongcun village

GPS track data reflect unevenly distributed tourist temporal-spatial behavior characteristics of different rural space types. As depicted in Fig. 5, the main tourist walkways, secondary tourist walkways, lanes and squares surrounding the heritage buildings and business streets were the most popular space areas in the village. However, other spatial regions displayed relatively lower kernel density values, indicating their lower popularity. Especially in E2, because there are only two historical trees in the village, the visiting proportion of tourists amounts to 83.15%, accompanied by an average dwell time of 3.08 min, which is almost half as much as that of E1 with the same spatial attribute, as shown in Table 4.

Regarding the characteristics of tourist temporal-spatial behavior, the study found that Visiting Proportion and Average Dwell Time have a strong correlation with other indicators, as shown in Table 7. It follows that they have a greater impact on tourist behavior variables.

**Table 7:** Correlation analysis between visitor behavior variables.

		Visiting proportion	Average dwell time	Average distance	Average speed
N		109	109	109	109
Visiting proportion	The correlation coefficient	1.000	0.758**	0.707**	− 0.269**
	Sig. (2 tails)		0.000	0.000	0.005
Average dwell time	The correlation coefficient	0.758**	1.000	0.959**	− 0.275**
	Sig. (2 tails)	0.000		0.000	0.004
Average distance	The correlation coefficient	0.707**	0.959**	1.000	− 0.121
	Sig. (2 tails)	0.000	0.000		0.211
Average speed	The correlation coefficient	− 0.269**	− 0.275**	− 0.121	1.000
	Sig. (2 tails)	0.005	0.004	0.211	

**At level 0.01 (2 tails), the correlation was significant.

*At level 0.05 (2 tails), the correlation was significant.

The non-parameter Spearman analysis shows a positive correlation among Visiting Proportion, Average Dwell Time and Average Distance, with the highest correlation observed between Average Dwell Time and Average Distance (Spearman’s ρ = 0.959, *p* = 0.000). Average Speed is negatively correlated with all three indicators, especially Average Dwell Time (Spearman’s ρ = -0.275, *p* = 0.004).

In Fig. 5, in spaces with high Visiting Proportion, the Average Dwell Time of tourists is longer, as well as the Average Distance. But the Average Speed drops, and vice versa. For example, in spaces B2, H1 and M1, the Visiting Proportion, Average Dwell Time and Average Distance are relatively high. However, the Average Speed is lower than in other spaces, indicating that business streets, heritage buildings and tourist walkways have a significant impact on tourist behavior. A8, C2, L5, and M3 with high Average Speed often suffer low Visiting Proportion due to their remote locations and lack of scenic spots worth visiting.

### Spatial patterns and characteristics of rural space in Hongcun village

1. Space syntax analysis illustrates that rural spatial attributes of a traditional village are differentiated by distinguishing features. The results of the Passable Layer reveal the accessibility of passable spaces, while the results of the Visible Layer reflect the attraction of space in the eyes of tourists. In the Passable Layer, squares and business streets have the highest value in spatial Connectivity, Control, Integration, and Intelligibility. On the contrary, the value of Mean Depth is lowest. In the Visible Layer, the values of those spaces, excluding the Hongcun Village central water, Yue Pond, and its surroundings, are relatively high. It shows that squares, business streets, water pools, and other spaces with open views have better accessibility than lanes and walkways (Fig. 8). For instance, B1, B2, E1, E2, and M2 are spaces with grander views than a narrow space. According to the Intelligibility analysis, the Passable Layer’s R^2^ score (0.400) was lower than the Visible Layer (0.518). It follows that in rural tourism, sight lines play a more dominant role in the guidance of tourists than accessible spaces.
  **Fig. 8:**
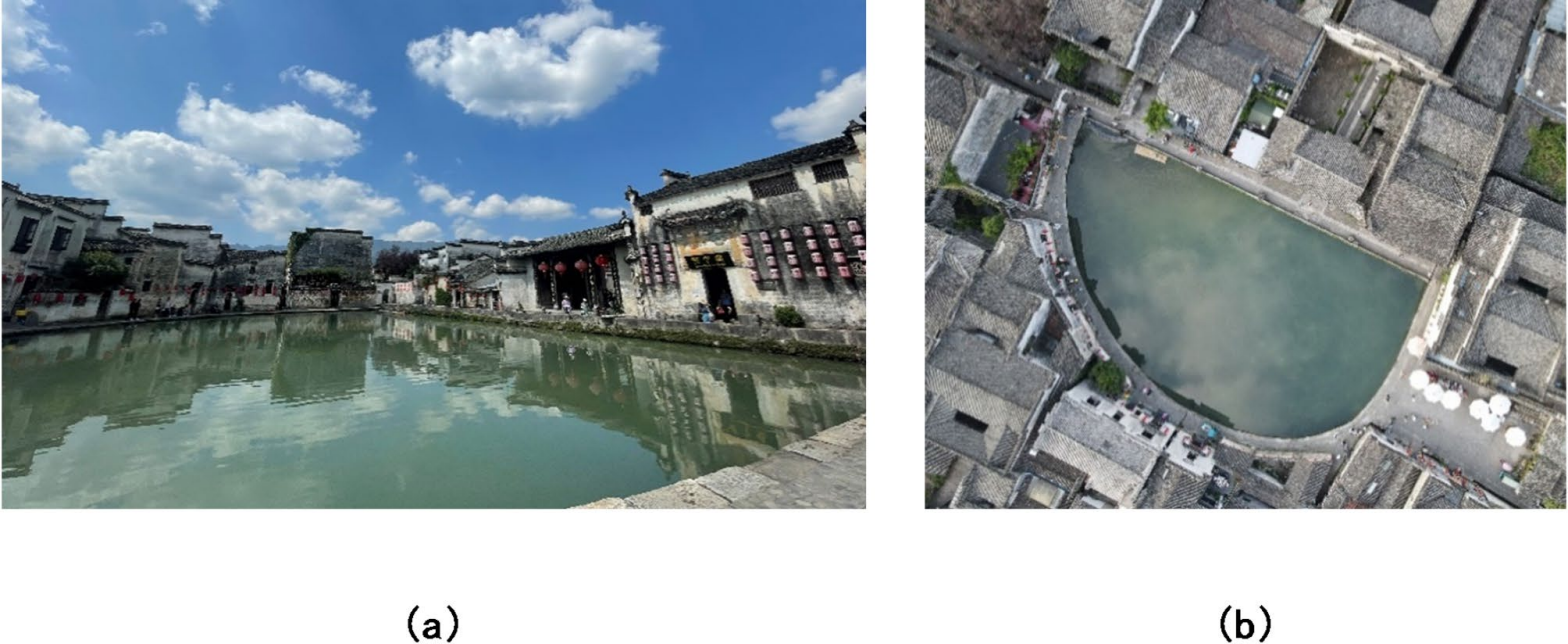
The photograph of Yue Pond. (**a**) photo of Yue Pond taken by camera; (**b**) photo of Yue Pond taken by drone.
2. Space syntax analysis demonstrates different spatial features when the same spatial attribute is applied at different layers. Analyzing the same space at different layers may result in different findings. Taking M2 for example, pertinent attribute values are shown in Table 8. The attribute values for M2 indicate a huge difference in Intelligibility. Compared to the Visible Layer (1036.59), the Passable Layer’s Intelligibility value (171.75) was significantly lower. This makes sense given that in M2, visual accessibility played an absolutely dominant role in this space because M2, the main tourist walkway, is centered around Yue Pond. Yue Pond, a water body in the center of the village, is not only located in the heart of Hongcun but also holds a profound cultural legacy and serves as one of the main scenic spots of the village. Further, it is surrounded by H1 (Wang family ancestral temple) and E1. The same relationship in Hongcun also includes E2.
  **Table 8:** Attribute values for space M2.

M2	The Passable Layer	The Visible Layer
Visiting proportion (%)	85.43	85.43
Average dwell time (s)	330.23	330.23
Average distance (m)	150.13	150.13
Average speed (m/s)	0.52	0.52
Connectivity	361.60	2248.81
Control	0.96	1.04
Mean depth	7.07	6.30
Integration	2.06	2.36
Intelligibility	171.75	1036.59

## Discussion

Rural tourism is significant for sustainable urban-rural development, which has notably become one of the most popular travel activities among city residents. This study focuses on the following results and we have contributed to the current rural tourism in the following ways: (1) Adopt mixed methods to obtain high-precision data, while most studies focus on a single-dimensional data acquisition method^77,78^; (2) Analyze the spatial morphological characteristics of rural areas through the examination of passable and visual layers, while most studies only focus on one aspect^79,80^; (3) Explore the correlation between tourists’ spatio-temporal behavior and spatial environment in Hongcun by collecting long-term tourist behavior data, while most studies focus on short-term tourist data perspectives^81,82^.

### Correlation characteristics of Spatial environment with tourist temporal-spatial behavior

As discussed before, space syntax analysis primarily focuses on the spatial characteristics of the space itself, while applying the findings of space syntax analysis to villages has certain limitations. That is, it cannot reveal tourist behavior characteristics of all rural space types. While some buildings can influence the behavior of tourists, GPS track data can accurately reflect the actual behavioral characteristics of rural tourists. Therefore, this research applies GPS track data, Weibo comments data, surrounding environment data and space syntax, conducting a data analysis that combines practice and theory.

Regarding the correlation characteristics of spatial morphology with tourist temporal-spatial behavior in Hongcun village, the study found that the Average Speed is independent of five variables of the Passable Layer, six variables of the Visible Layer and three variables of surrounding environment. On the contrary, Visiting Proportion, Average Dwell Time, Average Distance and Weibo comments have significant positive or negative correlation with the space syntax variables and surrounding environment variables. Tourists showed an obvious preference for staying in open spaces. The reasons for that may be the followings: (1) Easy space accessibility. Accessible spaces enhance the tourists’ experiences, leading to increased willingness among tourists to visit and stay. (2) Good visual permeability. A spacious space serves as a magnet for tourists from a distance. (3) Herd mentality. Most tourists will be attracted to crowded spaces, wondering why they are flocked with tourists.

Tables 5 and 6 suggest that the Visiting Proportion is shaped by spatial morphology to the greatest degree, as all space syntax variables affect tourists’ Visiting Proportion value in the space. This indicates that spatial morphology has a crucial impact on tourists’ decisions about whether to visit the space. It can be seen that the value of mean depth is negatively correlated to the Visiting Proportion in both Passable layer and Visible Layer. Mean depth represents the average distance between a certain space and all other spaces. The larger this value is, the more distance there is. It indicates that tourists have a significantly lower visiting proportion in remote spaces. The Weibo comments data is influenced by spatial morphology similarly to the Visiting Proportion. Among them, the passive layer is most closely related to Connectivity, while the visual layer is most related to Intelligibility. This indicates that the spatial location of tourists’ posts on social media is related to the convenience of transportation and the visual permeability.

Tourists’ Average Dwell Time and Average Distance value are also affected by spatial environment. However, it is weakly correlated to Mean Depth and Integration, indicating that accessibility and location are not the key factors in the process of tourists’ visits. Some spaces, like H2, Y1 and S10, are in remote places with inconvenient transportation or limited view. The former two are located along the Nan Lake, while H2 is a heritage building. There’s nothing special about their exterior, but the inside of the building is quite impressive. Y1 is one of the few forest spaces in the village. It is a courtyard inside H2, consisting of a platform, a corridor, and a public active area. Average Dwell Time of H2 and Y1 were 147.64s and 172.94s respectively. The latter consists of several intersecting lanes, which connects it to H4 and H5 and gives tourists the fun of passing between narrow lanes. For the above reasons, it can be inferred that tourists in the countryside prefer to stay in open spaces. Also, some spaces with less convenient transportation will attract tourists to their abundant and fascinating contents. NDVI shows a clear negative correlation with Visiting Proportion, Tourists Average Dwell Time, and Average Distance, indicating that areas with vegetation cover are not very attractive to tourists.

### Factors attracting tourists in rural tourism

However, how to improve the attractiveness of space in rural landscape to tourists and promote the sound and sustainable development of rural landscape in Hongcun remains an urgent problem. From the perspective of tourism management, fully utilizing and mobilizing rural resources is an important way to attract tourists and boost rural tourism^3^. Based on conducting a classification and summary study of rural landscape resources in Hongcun, and analyzing the relationship between tourist behavior and spatial environment, it is found that there are many ways to mobilize the utilization of rural landscape resources.

#### Visual permeability

As shown in Tables 5 and 6, whether in the Passable Layer or in the Visual Layer, syntactic variable most closely related to accessibility is Average Distance value which shows the more tourists move in the space, the more attractive and accessible the space is. Compared to other types of messages, such as scent, sound, and text. Visual information has a greater influence on people’s memories and attitudes^83^. The spatial sequence layout of Hongcun features “square -branch lane-gateway”. As the enclosing degree of space increases, it gives rise to a complete landscape sequence in which a tourist will see a different view with each step forward.

The spaces that have high Visual Clustering Coefficient values in Visual Layer have a high sight line limitation. In Table 6, the value of Visual Clustering Coefficient showed a remarkably negative correlation with Visiting Proportion, Average Dwell Time, and Average Distance, indicating that a space with a restricted view does not encourage tourists to stay and spend time sightseeing, while a space with an open view is more attractive to tourists. The higher the value of Intelligibility of one space, the more accessible the space in Passable layer is to other spaces, and the greater the Visual permeability of the space in Visible layer will be. Tourists visiting a space with high visual permeability will slow down the paces, stopping here and there to admire the captivating scenery. It reveals that spaces rich in visual information are more attractive to tourists.

#### Forest spaces

The ancient trees play a dominant role in influencing the attraction of tourists. The rural areas in southern Anhui are mainly homesteads with little forest space. Because E2 possesses the only two ancient trees in the village, it has a higher spatial Integration and Visual Clustering Coefficient than E1, as shown in Fig. 7. At the same time, the temporal and spatial behavior data of tourists reveal that the visiting proportion of tourists in E2 is 83.15% and the average dwell time is 185.17s, which is nearly half as much as that of E1. The results of them match. Furthermore, not only does the Visiting Proportion of E2 rank third in the whole village, surpassing E1, but it also comes second only to M1 and B1, which is sufficient to show the great influence of ancient trees on tourists’ behavior.

#### Heritage buildings and cultural legacy

Travel Destination is attempting to promote their cultural or heritage values for tourist consumption. Based on our observation, it is evident that not only are outdoor public spaces appealing to tourists, but historic buildings in the village are also a big draw for tourists, as shown in Fig. 5. Location H1, H2, H3, H4 are all heritage buildings. However, except for H1, the others are not located where visibility and accessibility are high. Among these heritage buildings, H3 stands out with the highest Average Dwell Time value (216.05s), despite not enjoying the best location. Unlike H1, which is in a space of high visibility and accessibility, H3 still attracts tourists. It follows that not only the wide vision is a draw for tourists, but the historical and cultural legacy of a space is an important factor in attracting tourists. It is crucial to reconcile the relationship between environmental protection and economic growth. Under the premise of ensuring the integrity of these cultural assets, we should fully express their cultural values and actively promote the development of rural cultural tourism.

### Effective rural landscape planning

This research prospectively explores the relationship between the behavior of visitors and different space scenes, and the results are as follows: (1) Easy space accessibility, good visual permeability, and herd mentality are the main reasons of forming uneven distribution of tourists; (2) Spaces rich in visual information are more attractive to tourists; (3) Historical trees, heritage buildings, and cultural legacy are the positive influencing cultural factors for tourist attraction in spaces.

East et al. conducted a study by tracking 931 groups of visitors and combining the tracking data with survey data to investigate whether various sorts of tourists acted differently during their visit to the destination. Similarly, the bulk of visitors traveled identical paths, indicating a strong ‘main path inertia’. However, this resulted in over half of the exhibits deviating from the perceived main route^84^. In Hongcun Village, most tourists are the same. Cultural tourism in rural areas is a significant factor in attracting tourists. Although it has been proposed that the community that manages cultural heritage believed that cultural tourism was a double-edged sword^85^. Based on the above discussion, the following rural landscape planning strategies can be used to mobilize the utilization of rural landscape resources of Hongcun village and improve the attractiveness of the space to tourists. (1) Balanced distribution of tourists to alleviate hypercongestion. For the unpopular spaces with low visibility and poor locations, enrich their cultural connotations to attract tourists; (2) Increasing the visibility of the spaces to reduce the restrictions on tourists’ view by the enclosed surroundings; (3) Increasing forest space, which can be built with ancient trees as a landscape focus; (4) There are no established tour routes in the village, with a maze of streets and lanes in all directions. we can guide tourists by adding signs. By adapting such measures, we can guide tourists to explore and visit the spaces, thereby significantly increasing the rate of space visits.

### Improvement in the research methods and data

In the context of the complexity of the geographical landscape and the lack of population data, this study uses mixed methods such as UAV, Python, GPS trajectories data, and space syntax, to collect high-accuracy data and quantitative data analysis. Surveys in rural areas are both time-consuming and labor-intensive, and there are issues with data incompleteness. The methods of questionnaires and handheld GPS data loggers, which are widely used in cities, are not suitable in rural settings. UAVs have been widely used in urban research but are rarely used in rural research. The primary reason is the complexity of rural landscapes, which makes it difficult for UAVs to plan routes for extensive aerial photography and flight study, unlike in metropolitan areas. UAVs typically need to fly manually in order to navigate complex terrain. Meanwhile, tourists and the spatial environment were considered and analyzed separately after integration. For tourists, trajectory data and Weibo comments data were integrated. For spatial environment, passive layer and visual layer were considered separately. In addition, rural landscape planning studies are still in a shortage, and many studies are focused on the human visual perspective, with most adopting a single-dimensional research approach, rarely paying attention to the tourists’ behavior. This study extends the perspective from the cities to the rural areas, from the human perspective to the spaces and high space perspective.

## **Conclusion**

### Theoretical implications

This study uses multi-data-mixed methods to quantify the relationship between tourist behavior and spatial morphology. It can be found that visual permeability plays a more dominant role in guiding tourists compared to spatial accessibility, while the accessibility of a space has the greatest impact on visiting rates. Accurate positioning of tourists’ spatial-temporal behavior characteristics by means of GPS track data indicates that the profound cultural legacy contained in the architecture and ancient trees in forest space can be highly appealing to tourists. An analysis of rural tourists’ preference for “hot spots” indicates that enhancing the comfort and guidance of spaces and making full use of the characteristics of the original space play a crucial role in future rural tourism planning.

### Practical implications

The findings of this study indicate that providing clear guidance, convenient transportation, and good spatial layout for tourists in rural tourism is extremely important. Rural managers and planners should pay more attention to how to better guide tourists in exploring the village. For example, we can set up signposts to direct tourists to visit public spaces or historical buildings that are not ideally located but are worth seeing. Moreover, the appeal of shops and restaurants to tourists cannot be ignored. Developing a good business environment is crucial for the development of rural tourism. We can develop a unique business street with local characteristics along the main road in the village to extend tourists’ visit time. Therefore, this study can contribute to the future planning and renovation of ancient villages, the preservation of their original cultural heritage, and the sustainable development of rural tourism.

### Limitation and recommendations

As a result, there are some limitations in the research process, which have led to the proposal of the following recommendations for additional study: The GPS trajectory data in this research was collected through cell phone localization. Cell phone signals can be interfered with by several factors, so the data thus collected may be somewhat unstable, coupled with some scattered GPS points in the original data. Also, due to the lack of data on the demographic characteristics of tourists, this research cannot conduct in-depth analysis on the behavior of tourists based on certain identities. In future research, we can use handheld GPS data loggers and mobile phone GPS data to enhance the precision of data. In addition, we can collect background information about tourists for each piece of data, including age, gender, education level, and occupation, to conduct targeted analysis of the behavior preferences of some tourists of specific identities. Depthmap can only analyze spatial morphology and visual features in two-dimensional space. Hopefully, future research can utilize other analysis methods, such as lidar, which enable a comprehensive stereoscopic analysis of spatial morphology in three-dimensional space through employing 3D modeling techniques. By processing tourist track data, we conducted a nuclear density analysis of tourist behavior, resulting in the acquisition of data on four variables related to tourist behavior. Using Depthmap to analyze the spatial morphology at Passable Layer and Visible Layer of Hongcun, we acquired related Depthmap’s data, respectively. Also, the correlation between each space syntax variable and four variables related to tourist behavior was calculated, and the decisive factors that make a space attractive to tourists in rural tourism were analyzed.

## Data Availability

The datasets generated during the current study are available from the corresponding author upon reasonablerequest.
